# Research and Innovations in Neurosciences

**DOI:** 10.12669/pjms.40.12(PINS).11419

**Published:** 2024-12

**Authors:** Shaukat Ali Jawaid

We are delighted to collaborate with the Punjab Institute of Neurosciences (PINS) to publish this special supplement, showcasing the exceptional research being conducted at their institution. Previously, we have published similar supplements for the Isfahan University of Medical Sciences in the Islamic Republic of Iran and the Indus Hospital and Health Network in Pakistan.^1^-^2^

This supplement includes two editorials, eleven original articles, six case reports, two systematic reviews, and three manuscripts in the correspondence section. We sincerely hope it contributes valuable information to global medical literature, particularly in the field of neurosciences from Pakistan.

This initiative began after a meeting in December 2023 with Prof. Asif Bashir, Executive Director and Dean of PINS, and one of the residents, Dr. Haseeb Mehmood Qadri. Following detailed discussions, the project was conceptualized, and a structured plan was devised to bring this vision to fruition. It was decided that Prof. Asif Bashir would serve as Guest Editor, while Dr. Haseeb Mehmood Qadri would act as Executive Editor for this issue, with the responsibility of coordinating with authors.

Once the manuscript drafts were received, they were subjected to plagiarism screening facilitated by the *Pakistan Journal of Medical Sciences*. The PINS team conducted initial screening and internal reviews before forwarding the manuscripts to subject-matter experts for external peer review. The reviewers’ comments were edited and communicated to the authors for revisions in line with the feedback. Finalized manuscripts were uploaded to the journal’s website along with essential documents, including a letter of undertaking, ethics committee approval, and two to three peer review reports. All manuscripts underwent at least one internal peer review and at least two external peer reviews.

The Pakistan Journal of Medical Sciences then downloaded these documents, added the plagiarism reports, and conducted an internal editorial review. The process of submission receiving, peer review and editorial review was closely and judiciously supervised by Secretary to Chief Editor PJMS. Accompanying documents were meticulously examined, and manuscripts underwent post-acceptance editing. During this process, challenges were identified in seven manuscripts, such as incomplete references, deviations from ICMJE authorship and citation guidelines, and discrepancies between in-text citations and reference lists. Additionally, some authors cited papers from predatory journals, which is against our publication policy. These references were removed or replaced, ensuring adherence to ethical standards.

After addressing these issues, formatted PDF files were generated and sent to the PINS team for proofreading. Following final corrections, DOIs were assigned, and the issue was published. This extensive process took almost a year to complete.

Dr. Haseeb Mehmood Qadri played a pivotal role throughout this project. Having earned a Certificate in Medical Editing and successfully managing this endeavor, he has emerged as a promising young editor and a valuable asset to PINS.

## The supplement features impactful research, such as:

- Complexity of arachnoid adhesions dictating the outcome of microvascular decompression for trigeminal by Tariq Imran Khokhar et al.^3^
- Incidence of Postoperative Cerebrospinal Fluid Leaks in Endoscopic Endonasal Transsphenoidal Surgery for Pituitary Adenomas by Sumira Kiran et al.^4^
- AXIEM-Guided Brain Lesion Biopsies by M. Aqeel Natt, providing crucial data from a low- and middle income country perspective.^5^
- DBS and Drug Reduction in Parkinson’s Disease by Zubair Mustafa and colleagues, emphasizing the efficacy of deep brain stimulation in early-stage disease.^6^
- Outcomes of Fenestration of Lamina Terminalis for Hydrocephalus following Clipping of Ruptured Aneurysms of Anterior Circulation by Ismaeel Khalid Iqbal.^7^
- Knowledge, Attitude, and Perception of Research Ethics among Postgraduate Residents by Haseeb Mehmood Qadri et al.^8^

Considerable valuable work is being undertaken at many medical institutions in Pakistan, yet much of it goes undocumented and unpublished for various reasons. However, when the head of an institution takes an active interest in academics and research, significant progress can be achieved. The publication of this supplement, showcasing research and innovations in neurosciences, serves as clear evidence of this potential.
